# Horizontal gene transfer of the *Pytheas* sequence from *Cuscuta* to *Orobanche* via a host-mediated pathway

**DOI:** 10.1038/s41598-025-31853-x

**Published:** 2025-12-11

**Authors:** Magdalena Denysenko-Bennett, Dagmara Kwolek, Grzegorz Góralski, Marek Szklarczyk, Renata Piwowarczyk, Saša Stefanović, Adam C. Schneider, Andrzej J. Joachimiak

**Affiliations:** 1 Department of Plant Cytology and Embryology, Institute of Botany, Faculty of Biology, Jagiellonian University in Kraków, 9 Gronostajowa St., 30-387 Kraków, Poland; 2 Department of Plant Biology and Biotechnology, Faculty of Biotechnology and Horticulture, University of Agriculture in Krakow, Al. 29-Listopada 54, 31-425 Kraków, Poland; 3 Center for Research and Conservation of Biodiversity, Institute of Biology, Jan Kochanowski University, Uniwersytecka 7 St., 25-406, Kielce, Poland; 4 Department of Biology, University of Toronto Mississauga, 3359 Mississauga Rd., Mississauga, ON L5L 1C6 Canada; 5 Department of Biology, University of Wisconsin–La Crosse, 1725 State Street, La Crosse, WI 54601 USA

**Keywords:** Evolution, Genetics, Plant sciences

## Abstract

Horizontal Gene Transfer (HGT) is a phenomenon of DNA transfer between organisms that does not involve a parent-offspring relationship. HGT is believed to play an important role in all groups of organisms, including plants, and especially in parasites. Here we describe a chain of transfers from *Cuscuta* (Convolvulaceae) to *Orobanche rigens* (Orobanchaceae)—both parasitic plants—via a host belonging to tribe Genisteae (Fabaceae). During its “journey” between genomes, the transferred sequence, which we named *Pytheas*, was altered by deletions, additions of new segments from the current genome, substitutions, and rearrangements. This is the first robustly documented case of a multi-step transfer pathway—involving one IGT and two HGTs—connecting three plant species. Specifically, this system involves a host plant mediated gene flow between two distinct parasites without involvement of transposable elements, the *cox1* intron, or other vectors. This case also demonstrates how host-parasite interactions can facilitate the spread of genetic material between evolutionarily distant lineages.

**Supplementary Information:**

The online version contains supplementary material available at 10.1038/s41598-025-31853-x.

## Introduction

Plant cells, in addition to the nucleus, contain two other types of organelles harboring their own genomes: mitochondria and plastids. However, their genetic material is not completely isolated, as DNA can be exchanged occasionally through a process called intracellular gene transfer (IGT), which is relatively common in flowering plants^1–4^. While IGT occurs within the same cell, another phenomenon called horizontal gene transfer (HGT) is responsible for the transfer of genetic material from one organism to another that is not its offspring^5,6^. Plant cell genomes vary in their receptiveness to foreign genetic material. For example, although both plastids and mitochondria have prokaryotic-type genomes, mitochondria have features that make them more acquiescent to foreign genetic material than plastids^7^. These include the capacity to actively import DNA and RNA across their membranes, the presence of long intergenic regions, an active homologous recombination system, and frequent fusion and fission events^7^. The mitogenomes of holoparasitic plants generally do not differ significantly from those of autotrophic angiosperms, except for a higher frequency of HGT events^8^. One of the most striking examples of massive HGT to the mitogenome of a holoparasite is *Lophophytum mirabile* (Balanophoraceae), which replaced most of its mitochondrial genes with those obtained from legume hosts^9^. Nevertheless, before drawing broader conclusions about mitochondrial genomes of holoparasites, it should be noted that data concerning these cases remain relatively limited^8^.

Possible DNA transfers from the plastid to mitochondrial genome(s) have been described, for example, in *Cuscuta*^10^. When integrated into the mitochondrial genome, plastid-derived DNA (mitochondrial plastid DNA – MTPT) has the potential to influence mitochondrial function by creating new promoters or gene forms, or by introducing novel functional tRNA genes. MTPT transfer can be attributed to either IGT or HGT. However, the plastid sequences are usually transferred intracellularly to the native mitochondrion before horizontal transfer^11^.

Horizontal transfer appears to be relatively common between parasitic plants and their hosts, where it is facilitated by the close physical contact of their tissues^7,12^. Furthermore, horizontal transfers between two parasites are possible. Based on a literature survey, only two cases of HGT between parasitic plant species have been reported to date: the transfer of the *ccmB* gene between two branch hemiparasites *Viscum album* and *Loranthus europaeus*^13^ and a possible transfer between *Orobanche* and *Phelipanche* of a plastid region containing the *rps2* and *rbcL* genes as well as the *trnL-trnF* region^14^. In both cases, HGT most likely occurred through parasite-to-parasite transfer, possibly via a single host simultaneously parasitized by both parasites, with no integration of the transferred DNA into the host genome.

A more probable scenario involves an intermediate host that receives and incorporates foreign genetic material from one parasite, then—after many generations or speciation events—serves as a donor when parasitized by a different holoparasitic species. Although HGT chains are known to occur, e.g., between bacteria, fungi, and plants^15^, such a situation has not been reported for plant species.

Here, we describe a sequential transfer chain in which a sequence originating from the *Cuscuta* plastid genome underwent intracellular gene transfer (IGT) to the *Cuscuta* mitochondrial genome; subsequently, it was transfereered horizontally (HGT) to the mitochondrial genome of a Genisteae species, and finally it was again transferred horizontally to the holoparasitic *Orobanche rigens*. This represents the first comprehensively documented multi-step transfer pathway linking three plant species through one IGT and two HGTs, in which a host plant mediates the genetic exchange between two distinct parasitic species.

## Results

**Fig. 1:**
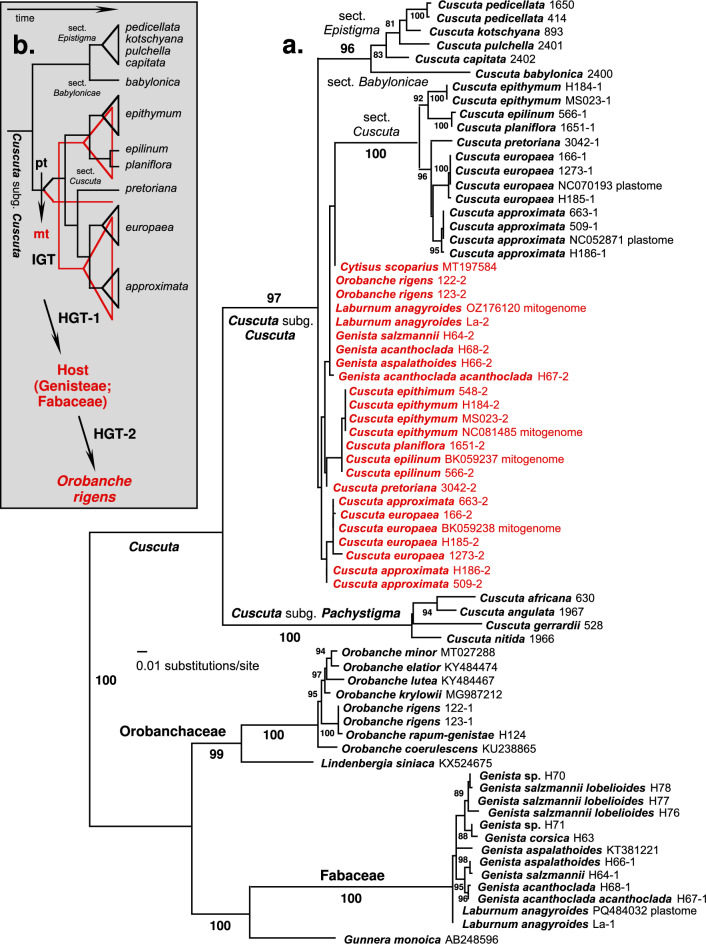
**a.** Phylogram resulting from maximum likelihood analysis of the plastid- (black) and mitochondrion-derived (red) *trnL-trnF* sequence data (-lnL = 7675.216). The tree is rooted using midpoint rooting, between *Cuscuta* (Convolvulaceae) and other included families (Orobanchaceae, Fabaceae, Gunneraceae) as functional outgroups. The maximum parsimony search resulted in a strict consensus tree (L=1236) with compatible topology. Bootstrap values (ML) are indicated for nodes supported at ≥80%; for additional values see Supplementary Figs. 2-3. Species names are followed by their DNA accession numbers (Supplementary Table 2). Suffixes −1 and −2 refer to those sequences inferred to be from plastid or mitochondrial genomes, respectively (compare with Supplementary Fig. 1). ‘Plastome’ and ‘mitogenome’ designate *trnL-trnF* sequences extracted from the entire plastid and mitochondrial genome sequences as deposited in GenBank. **b**. Schematic representation of evolutionary hypotheses derived from our phylogenetic (a) and other results (Supplementary Fig. 1, Supplementary Table 1). The initial event is inferred to be an intracellular gene transfer (IGT) of a *trnL-trnF*-containing DNA fragment from plastome (in black) to mitogenome (in red) that occurred in the common ancestor (stem lineage) of *Cuscuta* section *Cuscuta*. The next evolutionary step is the first horizontal gene transfer (HGT-1), from parasitic plants in this clade to their hosts in the Fabaceae tribe Genisteae. Finally, we infer an instance of secondary horizontal transfer (HGT-2), from the same set of hosts to *Orobanche rigens*. See text for further details and alternative explanations.

In *Cuscuta*, a double *trnL-trnF* amplicon resulting in a longer sequence (ca. 750–1000 bp) and a shorter sequence (ca. 400–500 bp) (Supplementary Fig. 1) was initially observed in a small number of samples from subg. *Cuscuta* sect. *Cuscuta* - *C. approximata*, *C. epithymum*, and *C. europaea*– used in broad-range phylogenetic studies (e.g^16,17^). This was not the case for many other samples from across the genus, including members from other sections of subg. *Cuscuta*. To build upon this observation, additional species were surveyed, including samples from all three sections of subg. *Cuscuta* (*Epistigma*, *Babylonicae*, and *Cuscuta*; sensu Costea et al. 2015^18^) as well as the closely related subg. *Pachystigma* (Fig. 1).

A similar phenomenon was independently observed in our previous phylogenetic studies on the *Orobanche* and *Phelipanche* genera^19,20^. We obtained a double *trnL-trnF* amplicon for two specimens of *O. rigens* from Sardinia and Corsica (Supplementary Fig. 1). The shorter PCR product was not detected in other samples of *Orobanche*, including *O. rapum-genistae*, which is regarded as the closest relative to *O. rigens* (see Discussion), or in *Phelipanche* species. Sequencing of the shorter PCR product from both *O. rigens* specimens yielded identical sequences of approximately 440 bp. This shorter sequence was subsequently used as a query in BLAST searches^21,22^. Results with 100−99% query cover included the mitochondrial genome of *Laburnum anagyroides* (100% identity), then the *trnL-trnF* region of *Cytisus* (Genisteae), mitochondrial and plastid genomes of several *Cuscuta* species, and *trnL-trnF* region of *Cuscuta* described as chloroplast sequence. Sequence similarity to the *Cuscuta* hits was notably higher for mitochondrial genomes than for plastid genomes. The top eight results, with 100-99% query cover, are shown in Supplementary Table 1. The *Cytisus* sequence (MT197584) is labeled in GenBank as "*Cuscuta* environmental sample isolate EDNA16-0043485 *trnL-trnF* intergenic spacer region, partial sequence; chloroplast". However, this sequence record includes a note stating that it was "purchased as *Cytisus scoparius*, Broom by TGoNS, 2016". We believe that the sequence was likely mislabeled due to its similarity to *Cuscuta*, and its original identification as *C. scoparius* is probably correct. Furthermore, it may represent a mitochondrial genome fragment.

*Laburnum* and *Cytisus* are closely related to *Genista*, and both classified in the Fabaceae tribe Genisteae. *Genista* contains three known hosts of *Orobanche rigens*: *G. corsica*, *G. salzmannii*, and *G. sulcitana*. Currently, the NCBI nucleotide database lacks mitochondrial or plastid genome sequences for these species. Sequences of the *trnL-trnF* region from *G. corsica* and *G. salzmannii* are present in the database, but they were not found in the BLAST results, nor were any other *Genista* sequences. Consequently, we conducted PCR screening of several *Genista* species, including two confirmed hosts of *O. rigens*. The additional PCR product was detected in single samples of *G. salzmannii*, *G. aspalathoides*, and *G. acanthoclada*. Conversely, this additional band was absent from two unidentified *Genista* samples from Sardinia and Corsica, *G. corsica*, and three samples of *G. salzmannii* subsp. *lobelioides*. The additional band was also observed in *O. rigens*, *L. anagyroides*, and two *Cuscuta* species (Supplementary Fig. 1).

To infer relationships among these gene copies, we built phylogenetic trees for the *trnL-trnF* gene using sequences newly obtained in this study as well as existing data from GenBank (Fig. 1a; Supplementary Figs. 2-3). Phylogenetic results from the plastid-derived *trnL-trnF* sequences (shown in black in Fig. 1a) are completely consistent with the results from broader phylogenetic analyses of these taxa^17,20,23^, both in terms of topology and support.

In *Cuscuta*, we observed a first split between subgenera *Pachystigma* (100% BS) and *Cuscuta* (97% BS). Within *Cuscuta* subgen. *Cuscuta*, three sections (*Cuscuta*, *Epistigma*, and *Babylonicae*) were found to be monophyletic and strongly supported, with relationships among their species matching those from previous studies^17,23^. Orobanchaceae, consisting of several *Orobanche* species and *Lindenbergia siniaca*, forms a separate sister clade (99% BS) to the clade formed by *Gunnera monoica* and Fabaceae (100% BS), in which *L. anagyroides* and the studied *Genista* species were included. The topology of the *Orobanche* branch is also consistent with previous studies^20^.

Nested within the plastid-derived *trnL-trnF* sequences from *Cuscuta* subgen. *Cuscuta*, were all of the mitochondrion-derived *trnL-trnF* sequences, including those obtained from *Cuscuta*, *O. rigens*, and the Fabaceae tribe Genisteae (shown in red in Fig. 1a). Overall, in the ML tree these sequences were recovered as paraphyletic with respect to the *Cuscuta* sect. *Cuscuta* clade, but the support for this paraphyly, as well as for the relationships among mitochondrion-derived *trnL-trnF* sequences, was weak, <80% BS and often <60% BS (Supplementary Fig. 2). Nonetheless, in the case of *Cuscuta* sequences, the results reflect the topologies obtained from plastid-derived sequences, albeit with very little support. The mitochondrion-derived sequences from the other two groups, *Orobanche* and members of the tribe Genisteae (Fabaceae), show no resolution at all (Fig. 1, Supplementary Fig. 2). Enforcing a topological constraint whereby monophyly of all mitochondrion-derived *trnL-trnF* sequences was imposed resulted in trees that were not significantly different from the ML trees when using Shimodaira-Hasegawa Test (ΔlnL = 3.563, p=0.259) or the Approximately Unibiased Test (p = 0.183). Together, these results are consistent with a single intracellular gene transfer (IGT) of the *trnL-trnF* sequence from plastome to mitogenome, inferred to have occurred in the stem lineage (common ancestor) of *Cuscuta* sect. *Cuscuta* clade (Fig. 1b).

The parsimony analysis resulted in more than one million equally parsimonious trees (length = 1236; consistency index = 0.753; retention index = 0.943). The strict consensus of the MP trees (not shown) is topologically fully compatible with the ML tree (Fig. 1a), with one notable exception. According to the parsimony results, all sampled mitochondrion-derived *trnL-trnF* sequences in our data are found in a single clade, supported with 91% parsimony BS (Supplementary Fig. 3) and sister to the *Cuscuta* sect. *Cuscuta* clade. This result further supports the single intracellular transfer of the *trnL-trnF* sequence from plastome to mitogenome.

Our results appeared to confirm the occurrence of HGTs; however, we sought further information regarding the length of the transferred sequence in *Orobanche rigens*. To address this question, we aimed to extend the PCR-amplified fragment. As mentioned above, the investigated sequence was found in *Laburnum anagyroides* which is related to *Genista*, and its mitogenome is available in GenBank (acc. no. OZ176120). Therefore, we used it to design a series of PCR primers (Supplementary Table 3) to amplify the potentially longer transferred sequence in *O. rigens*. Using these primers, we obtained a sequence of 5076 bp. Similarly, a 5114 bp sequence for *G. salzmannii* (specimen from Sardinia) was obtained. The positions of primers on the *L. anagyroides* sequence and PCR products obtained for *O. rigens* and *G. salzmannii* are shown in Supplementary Fig. 4.

Surprisingly, comparison of the homologous sequences revealed that the *O. rigens* sequence was more similar to *L. anagyroides* than *G. salzmannii*. The differences between *O. rigens* and *L. anagyroides* were 54 indel sites and 16 substitution sites, while the differences between *O. rigens* and *G. salzmannii* were 61 indel sites and 21 substitution sites. For this reason, *L. anagyroides* was selected for further analyses.

**Fig. 2:**
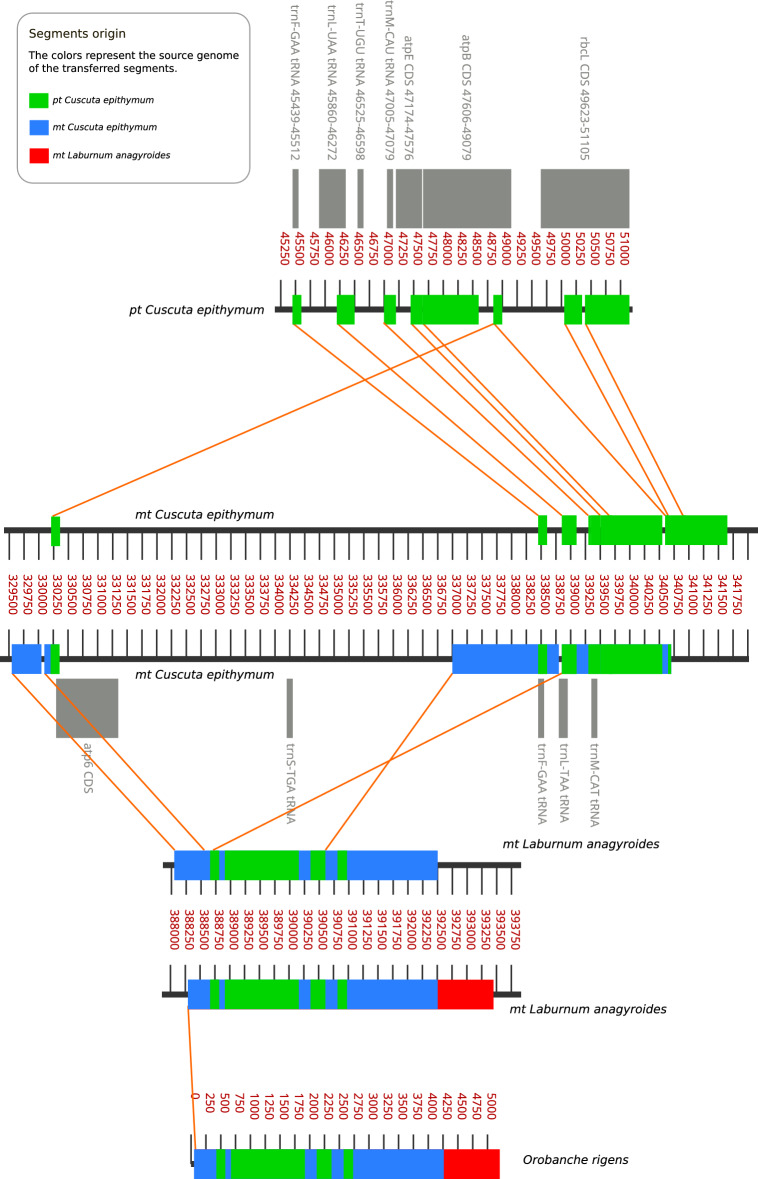
Segments aligned between genomes of plastid *Cuscuta epithymum*, mitochondrial *C. epithymum*, mitochondrial *Laburnum anagyroides*, and the sequence of *Orobanche rigens*. Colors of the segments indicate the genome from which they originate. Each sequence consists of parts originating from the genomes through which the sequence was transferred: first from the *Cuscuta* plastid, then from *Cuscuta* mitochondria, and finally from *Genisteae* species. All identified carriers left a “fingerprint” — sequences characteristic of a given genome/taxon — allowing their identification. These “fingerprints” indicate the direction of transfers. While the exact *Cuscuta* species involved in the transfer was a common ancestor of multiple extant *Cuscuta*, we use *C. epithymum* as a model because it was the only candidate species with both plastid and mitochondrial genomes sequenced.

The BLAST search using the 5076 bp *Orobanche rigens* sequence as a query revealed that the most similar sequence was from *L. anagyroides* (100% cover, 98.52% identity). Further results, ordered by query cover, included sequences from mitochondrial genomes of three *Cuscuta* species - *C. europaea*, *C. epithymum* and *C. epilinum* - with 81% cover and 92.80−90.57% identity. The next result was from the mtDNA of *Genista pilosa* (72% cover, 98.46 identity). Other results had at most 66% cover. The highest-ranked plastid genomes of *Cuscuta* species (by cover) - *C. pedicellata*, *C. europaea*, *C. epithymum* and *C. approximata* - reached positions 7–10, having 40–42% cover and 93–95% identity. Next, we performed a series of local BLAST searches using organelle genomes of *Cuscuta* species and *L. anagyroides* available in GenBank, and the sequence obtained for *O. rigens*. The graphically presented results of sequential local BLAST alignments and their interpretation are shown in Fig. 2.

The alignment of the 5076 bp *O. rigens* sequence to the mitochondrial genome of *L. anagyroides* resulted in a main alignment of 5127 bp (region: 388267–393389) with 98.5% identity and 55 gaps. More detailed comparison of the alignment revealed that the *O. rigens* sequence had lost several short segments and that there were some possible minor insertions. The aligned sequence from *L. anagyroides* mtDNA was then locally aligned by BLAST to the mitochondrial genome of *C. epilinum*. The sequence studied was aligned from the beginning to 4205 bp; however, the alignment with *C. epithymum* (region 329801–340618) was divided into two main segments, one short (516 bp) and one much longer (3639 bp). Both segments were divided into two smaller parts (but closely located—less than 100 bp) and were separated by 6662 bp and inverted relative to each other. It is worth noting that the part of the mitochondrial genome of *C. europaea* related to the larger segment had annotations indicating plastid origin and included *trnM*, *trnL,* and *trnF* genes^24^.

Local BLAST results of the mitochondrial genome of *C. epithymum* (and the part that was identified as a source of HGT to *Laburnum*) against the plastid genome of the same species show several aligned DNA fragments divided by non-aligned sequences, which are generally shorter in mtDNA than in ptDNA. In the plastid, the segment containing aligned fragments includes various important coding sequences such as *trnF*, *trnL*, *trnT*, *trnM*, *atpE*, *atpB*, and *rbcL*. Alignment analysis revealed that portions of the *O. rigens* sequence are highly similar to plastid genes, including *trnF* (100% identity), *trnM* (97%), and *trnL* (91%). Notably, the *atpB* and *atpE* gene sequences also contain some sections with low differentiation between the plastid genes and the mtDNA of *O. rigens.*

## Discussion

*Orobanche rigens*, in which the double *trnL-trnF* amplicon was found, is endemic to Sardinia and Corsica (also reported from Sicily, possibly in error) and is associated only with island endemic hosts of woody Fabaceae: *Genista corsica*, *G. salzmannii*, and *G. sulcitana*. Some authors include *O. rigens* as a subspecies of the more widely distributed *O. rapum-genistae*. However, the latter differs in some morphological features as well as host range—especially *Cytisus scoparius*, rarely *C. striatus*, *C. multiflorus*, other *Cytisus* species, and *Genista*^25^. The absence of the additional band for the *trnL-trnF* amplicon in *O. rapum-genistae* supports the view that it is a separate species.

The BLAST search using the additional PCR product of *O. rigens* as a query found that the most similar sequences were the *Laburnum anagyroides* mitochondrial genome and *Cytisus scoparius* sequences. These two species belong to the tribe Genisteae in the family Fabaceae and are related to the genus *Genista*, which contains hosts of *O. rigens*.

In subsequent positions, the BLAST results indicated mitochondrial and plastid genomes of *Cuscuta* species. Considering that the sequence studied contains the *trnL-trnF* region, these results suggest that it was initially part of the *Cuscuta* plastome and was then transferred to the mitogenome via IGT.

**Fig. 3:**
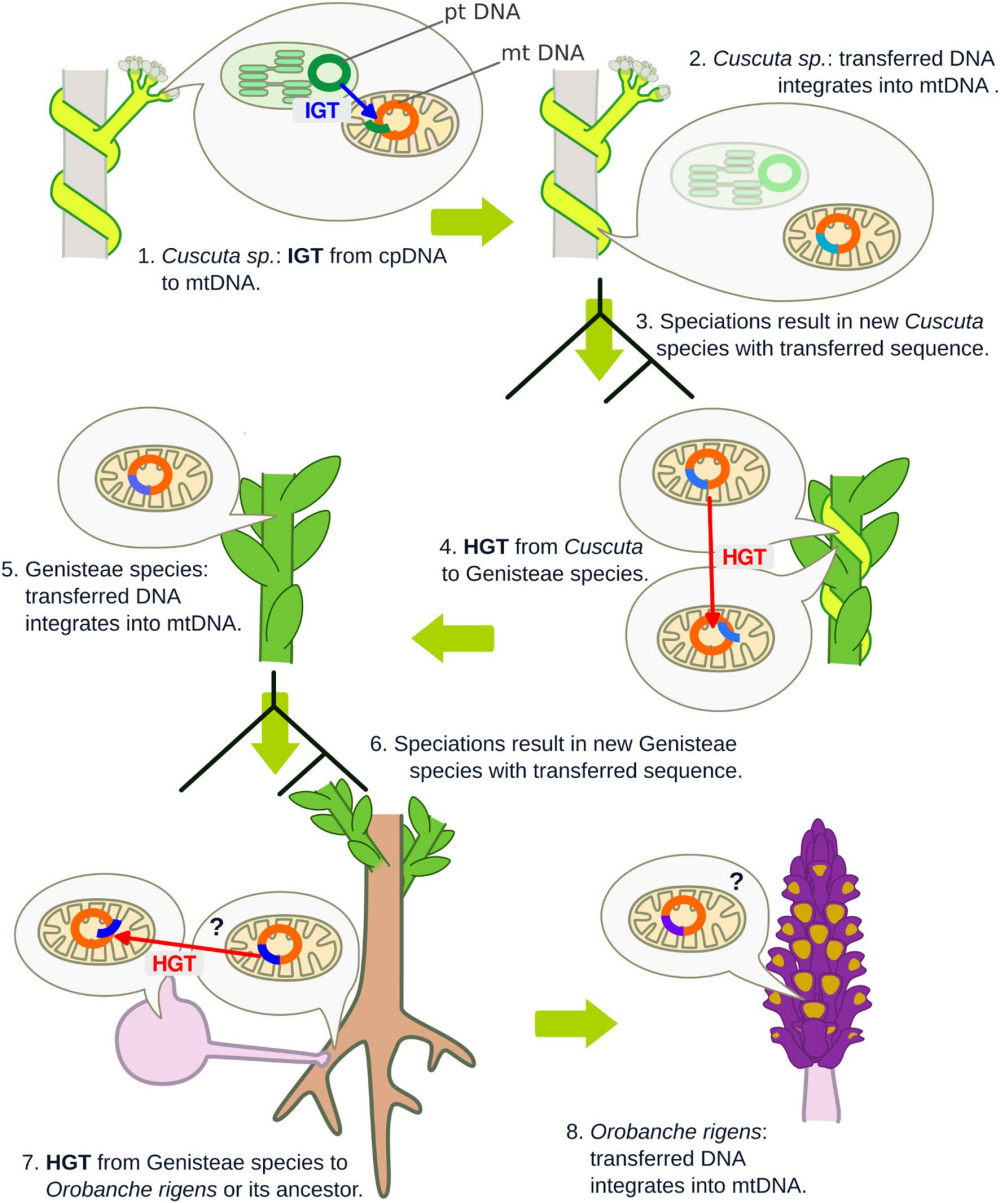
The proposed pathway of *Pytheas* transfers from *Cuscuta* plastid to the mitochondrion of *Orobanche rigens*. Changing colors of the transferred sequence indicate changes during the process, including different parts of the sequence transferred, point mutations, insertions, deletions, and rearrangements of segments (compare with Fig. 2).

Based on our results, we formulated the hypothesis (Fig. 3) that the studied sequence, which we named “*Pytheas*” after the ancient Greek explorer and which includes the *trnL-trnF* region, originated from the plastid genome of an unidentified *Cuscuta* species. Through IGT, it was transferred from the plastid to the mitochondrial genome within *Cuscuta*. Then *Pytheas* was transferred via HGT to the mitochondrial genome of an unknown *Cuscuta* host, which probably belonged to the tribe Genisteae. This host was an ancestor of several *Genista* species and related species, including *Laburnum anagyroides*. In the next step, *Orobanche rigens* (or its ancestor) received *Pytheas* from its host, also via HGT, and incorporated it into its mitochondrial genome. Like an intrepid explorer, at each stage of its journey, *Pytheas* was slightly altered, including deletions, insertions, and rearrangements. Importantly, it accumulated genomic material from each stage of its journey. These became vestiges that we were able to use to reconstruct its journey across cellular and species boundaries (Fig. 3).

We cannot precisely identify the primary or secondary donor species that served as vectors for this genome fragment in the multistep transfer process. Consequently, precise characterization of the transferred sequence and its modifications is limited by incomplete mitochondrial and plastid genome databases and the possibility that donor species are now extinct. Therefore, we reconstructed the process using the most similar sequences available in NCBI databases. For the process summary (Fig. 3), we selected *C. epithymum* as it was the only candidate species with both plastid and mitochondrial genomes sequenced. Despite these limitations, we believe our reconstruction accurately depicts the IGT and HGT flow from a *Cuscuta* plastid to the *O. rigens* mitochondrion.

The results also suggest that the segment transferred from plastid to mitochondrion in an unidentified *Cuscuta* species was initially much longer but later lost significant parts of the sequence and was rearranged. Other (than that of *C. epithymum*) tested *Cuscuta* mitochondrial genomes (*C. epilinum* and *C. europaea*) displayed a similar alignment pattern, but there were differences regarding, for example, separation of the segments and their orientation. Moreover, the true HGT donor might have had the sequence arranged in a way more similar to that observed in *Laburnum*. It is worth noting that the part of the mitochondrial genome of *C. europaea* related to the larger segment had annotations indicating plastid origin genes^24^.

After IGT from the plastid to the mitochondrion, these fragments evolved independently in both genomes. This transfer was the oldest of those described, so the expected number of changes between the plastid sequence and others is the highest. The question is why some segments appear much more conserved than the blocks between them. The alignments of these plastid genes to the *O. rigens* sequence show that some transferred genes may maintain this activity due to their high sequence identity; these include *trnF* (100% identity), *trnM* (97%) and *trnL* (91%). Importantly, the *atpB* and *atpE* gene sequences also contain some sections with differentiation between plastid genes and the mtDNA of *O. rigens*.

It is worth emphasizing that the scenario described above, involving a three-step pathway from the plastid of *Cuscuta* to *O. rigens* mitochondrion, should not be treated as a definitive sequence of events revealed by our studies but rather as the most parsimonious interpretation of the available data. However, several arguments support this hypothesis. The direction and order of transfers are supported by the presence of plastid sequences (which indicate the origin of the sequence) and flanking sequences, which act as “signatures” of the donors. Furthermore, the lowest rate of differences is observed between *L. anagyroides* and *O. rigens*, and the sequence studied was found in only one *Orobanche* species (it was absent even in the closest species *O. rapum-genistae*). These observations support the view that the HGT from Genisteae to *O. rigens* was the last HGT event, at least among those identified.

The bottom drawing in Fig. 2 illustrates the *Pytheas* structure in *O. rigens* with regard to the origins of its segments. It shows that the sequence consists of parts originating from the genomes through which the sequence was transferred: first from the *Cuscuta* plastid, then from *Cuscuta* mitochondria, and finally from Genisteae species. All identified carriers left a “fingerprint” — sequences characteristic of a given genome/taxon — allowing their identification. These “fingerprints” indicate the direction of transfers.

Theoretically, other possible scenarios exist for HGT from *Cuscuta* to *O. rigens*. Hyper- or epiparasitism—where a parasite exploits another parasite^26^—has been reported in several instances. Notably, *Cuscuta* species have been observed parasitising other parasites, including conspecific *Cuscuta* individuals and hemiparasitic members of the Orobanchaceae and Santalaceae families^26,27^. Moreover, a case of double parasitism has been observed, in which *Genista* sp. (Fabaceae) is infected by both *Cuscuta* sp. and *Phelipanche* sp. (Orobanchaceae) simultaneously^26^. The fifth author (R.P.) observed *Cuscuta* directly parasitizing holoparasites from the *Orobanche* and *Phelypaea* genera (unpublished data). Such observations may suggest that *Pytheas* could have been transferred from *Cuscuta* to *O. rigens* directly or via *Genista*, on which both parasites parasitized simultaneously. However, such a possibility can be rejected, mainly because in the *O. rigens* genome, *Pytheas* contains segments of *Genista* (or its relative) DNA origin, which indicates that the sequence was in the interim integrated into the host’s genome. Moreover, this does not explain the presence of the sequence in different Genisteae species (Fig. 1a).

Notably, there are known cases of horizontal transfer of mitochondrial genes from *Cuscuta* to hosts^28–30^ but the first known example of mitochondrial gene HGT (*atp1*) to *Cuscuta* from its host was reported in 2022^31^, so it is probably a rare phenomenon. This may be explained by the relatively small size of *Cuscuta* mitogenomes, which creates a barrier to including foreign sequences in their mtDNA^10^.

The presence of *Pytheas* in many *Cuscuta* species indicates that the IGT occurred in their ancestor. Similarly, it was found in many Genisteae species, even in different genera (*Genista*, *Laburnum*), meaning transfer to a common ancestor is probable. *Pytheas* was not found in all studied *Genista* species, which can be explained by different scenarios. This may indicate degeneration or loss of the sequence in some evolutionary lines of Genistae. Another possibility is the phenomenon of incomplete lineage sorting - *Pytheas* was present in some but not all individuals of the ancestral population, so when it diverged into different evolutionary lineages, only some of them inherited the transferred sequence.

The similarity of the sequence in *O. rigens* and *L. anagyroides* is particularly striking. The second sequence from a Genisteae tribe plant that aligned by BLAST to the *Pytheas* sequence from *O. rigens* (5076 bp) was from *Genista pilosa*, but it had much lower coverage (72%) than *L. anagyroides* (100%). The *G. salzmannii* sequence obtained during our studies is more similar but still less similar than that of *L. anagyroides*. The lack of data regarding mitochondrial genomes of more *Genista* species also limits our conclusions. It cannot be excluded that on exceptional occasions, *O. rigens* or its ancestor could parasitize other species, potentially more closely related to *L. anagyroides* than its current hosts. Alternatively, the transferred sequence might be generally quite conservative in Genisteae, but the mitogenome of *G. pilosa* may have lost part of the sequence.

The genomic location of the sequence found in *O. rigens* is uncertain; however, mitochondrial residence remains the most plausible, given the well-documented occurrence of mitochondrion-to-mitochondrion transfers^7^. The HGT involvement of mitochondria is facilitated by their propensity to fusion events^32^ as well as the extreme organizational plasticity of plant mtDNA^33^. Extreme examples of HGT between mitochondria include parasitic Rafflesiaceae in which up to 41% mitochondrial gene sequences showed signs of host origin^34^, and *Amborella trichopoda*– its mitochondrial genome was found to contain the entire transferred mitochondrial genome of the moss *Anomodon*^35^. Given the propensity for mitochondrial fusion and rearrangements, our phylogenetic analysis (Fig. 1) and BLAST comparisons (Fig. 2), the mitogenome is the most probable location of the *Pytheas*. Furthermore, the low support and relatively short branch lengths in these sequences, compared to their plastid-derived counterparts, correlate with the substantially lower point mutation rates generally observed in plant mitochondrial genomes^36–38^. In conclusion, HGT of *Pytheas* involving the nucleus or plastid is extremely unlikely.

All these results support our hypothesis of a three-step transfer of the *Pytheas* sequence. This is an interesting example of the long-range travel of *Pytheas* between different types of genomes and taxa. Finally, it may represent a fascinating new example of the role of parasites in spreading genes between evolutionary lineages.

## Materials and methods

### Plant material

Fresh and herbarium-obtained plant material used in this work is listed in Supplementary Table 2. Most of the plant material samples were obtained from herbaria. Experimental research and field studies on plants, including the collection of plant material, complied with relevant institutional, national, and international guidelines and legislation, and necessary permits were obtained. The material collected for this work has been deposited in publicly available herbaria.

Fabaceae (tribe Genisteae) species used here represent different *Genista* species collected in various localities, including two of the three known hosts of *Orobanche rigens*. In the case of *Laburnum anagyroides*, the mitochondrial genome sequence was downloaded from GenBank, and we obtained additional mitochondrial sequences from a commercially sourced (Szkółka Pnączy Wędrowski, Poland) plant specimen.

*Orobanche rigens* (Orobanchaceae) was collected in Corsica (France) and Sardinia (Italy), two islands where this species is endemic. Samples of *O. rapum-genistae* originate from Andalusia (Spain). Sequences of other *Orobanche* species were deposited in GenBank during our previous phylogenetic studies of Orobanchaceae^19,20^.

Moreover, multiple individuals from the *Cuscuta* sect. *Cuscuta* (Convolvulaceae), representing a broad geographical range, were included in this study. In addition to three DNA samples used previously, total genomic DNA was isolated from 19 plants representing new localities – this material was obtained from herbaria and field collections. Additionally, five more samples were represented by sequences from GenBank – the region of interest was extracted from the complete plastomes or mitogenomes of closely related *Cuscuta* species (Supplementary Table 2). These latter sequences were included in our analyses as phylogenetic ‘anchors’ for the organellar location of our ‘double amplicon’ sequences (Supplementary Fig. 1).

The sequence of *Gunnera monoica* was downloaded from GenBank.

### Molecular techniques

#### Orobanche and Genisteae specimens

Total DNA was extracted using the GeneMATRIX Plant & Fungi DNA Purification Kit (EURx) according to the manufacturer’s protocol. The plastid DNA *trnL-trnF* region consisting of the tRNA-Leu (*trnL*) intron, the *trnL* 3’ exon, and the intergenic spacer between the latter and tRNA-Phe (*trnF*) coding sequence was amplified using primers *c* and *f*^39^ as described in Kwolek et al.^40^. When double bands appeared, the amplicons were separated by agarose gel electrophoresis before purification and sequencing. The mitochondrial genome sequence of *Laburnum anagyroides* (acc. no. OZ176120), the species related to *Genista* in which the studied sequence was found, was used to design a set of additional primers. The primers that were implemented for sequencing and amplifications are detailed in Supplementary Table 3. The locations of primers and expected PCR product lengths for the *L. anagyroides* mtDNA segment are shown in Supplementary Fig. 4. In the case of *O. rigens*, PCR products were obtained for pairs of primers: *La-F1* and *La-R1*, *La-F4* and *La-R4*, *La-F5* and *La-R7*, *La-F8* and *La-R7*, *La-F9* and *La-R7*, *La-F9* and *La-R1*, *La-F11* and *La-R13*. Each amplicon was sequenced with the primers used for its amplification. For the *La-F1* and *La-R1* product, *La-F2*, *La-F3*, *La-R2*, *La-R3*, *f*, and *c* were also used for sequencing as internal primers.

PCR products for *G. salzmannii* were obtained using the following pairs of primers, with additional internal sequencing primers listed in parentheses: *La-F1* and *La-R2* (*La-F2*), *La-F2* and *La-R3* (*La-F3*, *La-R2*,* f*), *La-F3* and *La-R1* (*La-F9*, *La-R3*), *La-F4* and *La-R4*, *La-F5* and *La-R13*, *La-F8* and *La-R14*, *La-F14* and *La-R15*, *La-F15* and *La-R16*. The newly generated sequences have been submitted to GenBank (accession numbers PV940715-PV940730, PX020891-PX020900; see Supplementary Table 2).

#### Cuscuta specimens

For *Cuscuta*, DNA extraction and purification, PCR reagents and conditions, as well as amplicon purification and sequencing followed the protocols detailed in Costea and Stefanović^19^. In those cases where a double band was obtained, amplicons were separated from each other via agarose gel before further purification and sequencing. Sequences newly generated for this study were deposited in GenBank (accession numbers PX514516-PX514540; see Supplementary Table 2).

### Bioinformatic methods and phylogenetic inference

For local BLAST alignments and their graphic representation, an in-house software BlastAndShow (https://github.com/ggoralski/blast_and_show) was created using Python and necessary libraries, e.g., Biopython^41^. Searches of similar sequences in NCBI databases were performed using the BLAST tool^22^ and the ‘Nucleotide collection (nr/nt)’ database.

Sequences were initially aligned automatically using MAFFT^42^ and then manually adjusted using Se-Al v.2.0a11 (http://tree.bio.ed.ac.uk/software/seal/). Although numerous gaps had to be introduced in the alignments, the sequences were alignable across various taxa as well as among plastid- and mitochondrion-derived *trnL-trnF* sequences. Regions that could not be unambiguously aligned were excluded from subsequent analyses. Gaps in the alignments were treated as missing data. Phylogenetic analyses were conducted under maximum likelihood (ML) and maximum parsimony (MP) criteria.

Maximum likelihood analyses were performed using RAxML-HPC2 v.8.2.10^43^ and run on the XSEDE computing cluster using the CIPRES Science Gateway v.3.3^44^. ModelFinder^45^ was used to determine the model of sequence evolution that best fit the data. We used the same model of sequence evolution (TVM + G + I) and 1,000 rapid bootstrap replicates to assess branch support.

In parsimony searches, nucleotide characters were treated as unordered, and all changes were equally weighted. In these analyses, searches for most parsimonious trees were performed with PAUP* v.4.0b10^46^, using a two-stage approach. The analyses first involved 100,000 replicates with stepwise random taxon addition, TBR branch swapping saving no more than 10 trees per replicate, and MULTREES off. The second round of analyses was performed on all trees in memory with the same settings except with MULTREES on. Both stages were conducted to completion or until one million trees were found. Support for clades was inferred by nonparametric bootstrapping^47^, using 500 heuristic bootstrap replicates, each with 20 random addition cycles, TBR branch swapping, and the MULTREES option off.

To investigate an alternative topological hypothesis, we constructed a constrained tree by imposing monophyly of all mitochondrion-derived *trnL-trnF* sequences (red in Fig. 1). To statistically test and compare this enforced monophyly with the optimal ML tree, two statistical tests were conducted under the maximum likelihood criterion: the one-tailed Shimodaira-Hasegawa test (SH test^27^) and the Approximately Unbiased test (AU test^28^), using 10,000 resampling with the RELL method^29^ and full parameter optimization of the model in PAUP* v.4.0b10^46^.

## Supplementary Information

Supplementary Information.

## Data Availability

The datasets (DNA sequences) generated and/or analysed during the current study are available in the USA NIH Genbank repository, https://www.ncbi.nlm.nih.gov/genbank/. We declare that all data on the basis of which this manuscript was created are publicly available and disseminated in the manuscript itself or as supplementary materials.
