# Supplementary material for: Horizontal gene transfer of the Pytheas sequence from Cuscuta to Orobanche via a host-mediated pathway
Source: Sci Rep. 2025 Dec 11;16:2056. doi: 10.1038/s41598-025-31853-x (PMC12808232; doi:10.1038/s41598-025-31853-x)
Supplement: Supplementary file 1 — Supplementary Information. [file 41598_2025_31853_MOESM1_ESM.docx]

# Supplementary figures and tables

## Figures


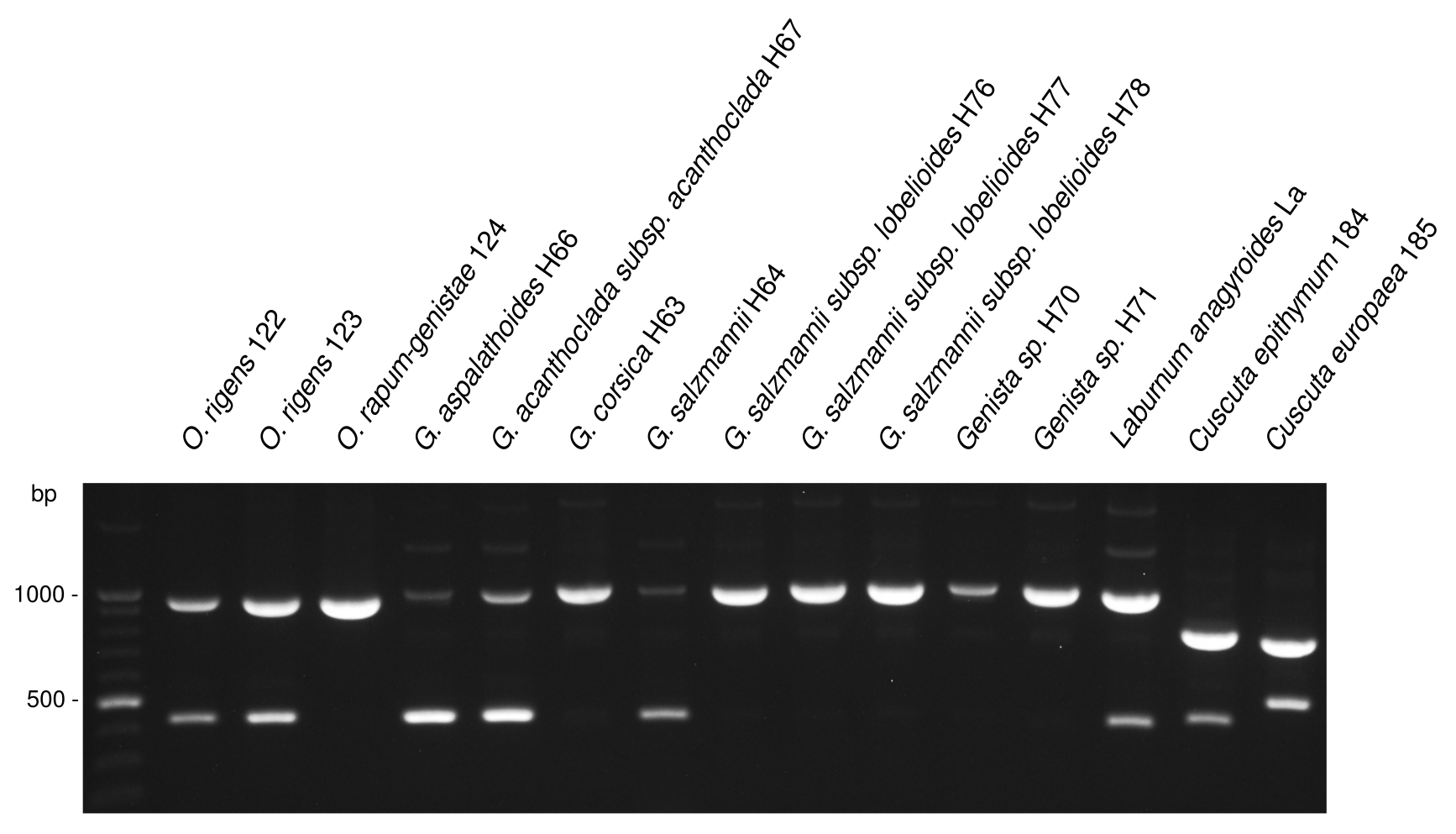


**Supplementary Figure 1.** PCR products obtained using primers c and f for a representative selection of studied species of *Orobanche*, *Genista*, *Laburnum*, and *Cuscuta*. Note the presence of double *trnL-trnF* amplicons in some species, resulting in one longer sequence (ca. 750-1000 bp) and one shorter sequence (ca. 400-500 bp).


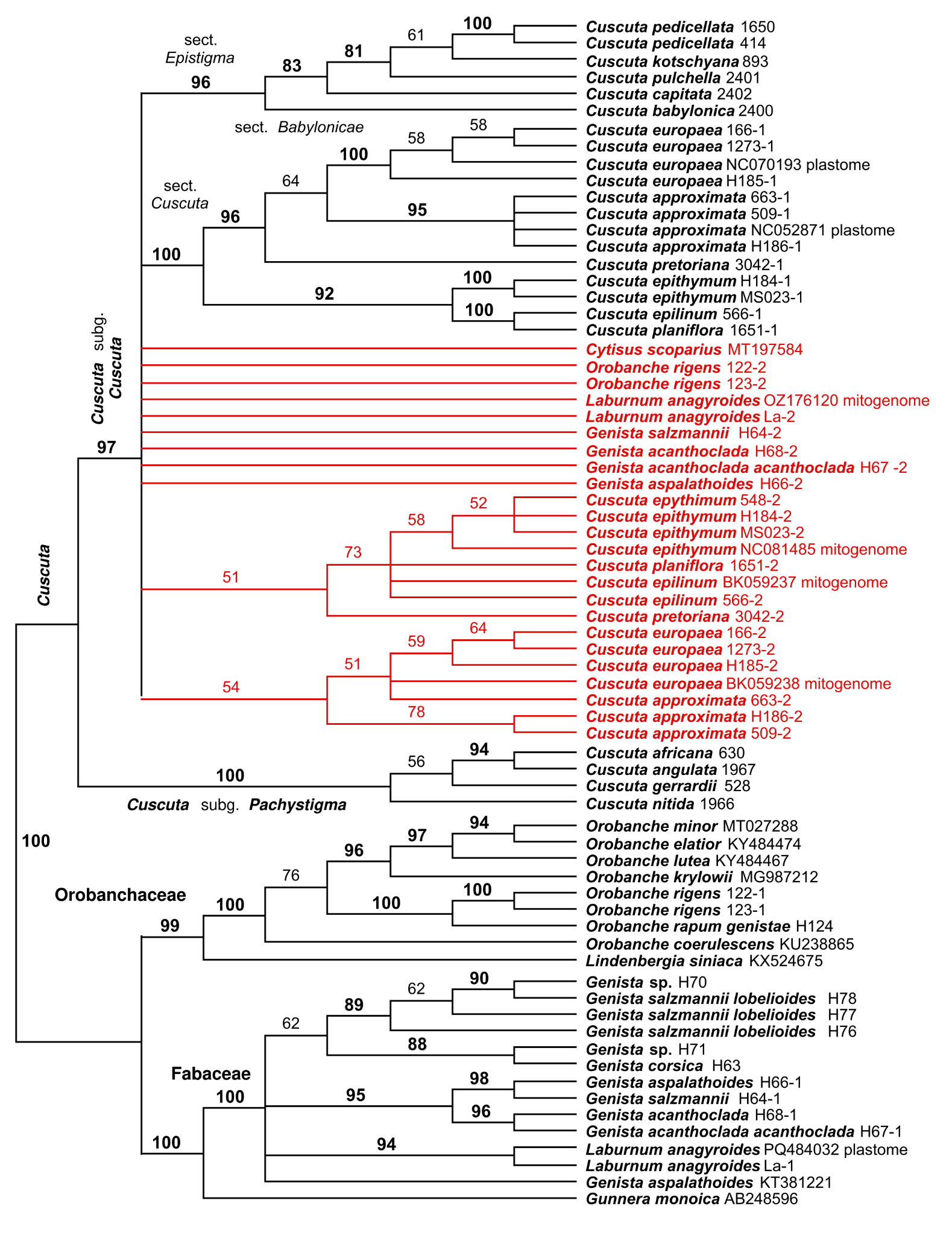


**Supplementary Figure 2.** Bootstrap consensus tree resulting from 1,000 rapid bootstrap replicates under maximum likelihood optimality criterion (see text for details of ML analyses and models of DNA evolution used). Bootstrap values are indicated for nodes supported at ≥50%. Highlighted in bold are those that are ≥80%, as shown in Fig. 1. Species names are followed by their DNA accession numbers (Supplementary Table 3). Suffixes -1 and -2 refer to those sequences inferred to be from plastid or mitochondrial genomes, respectively. ‘Plastome’ and ‘mitogenome’ designate *trnL-trnF* sequences extracted from the entire plastid and mitochondrial genome sequences as deposited in GenBank. Compare with Fig. 1 for further details and interpretations.


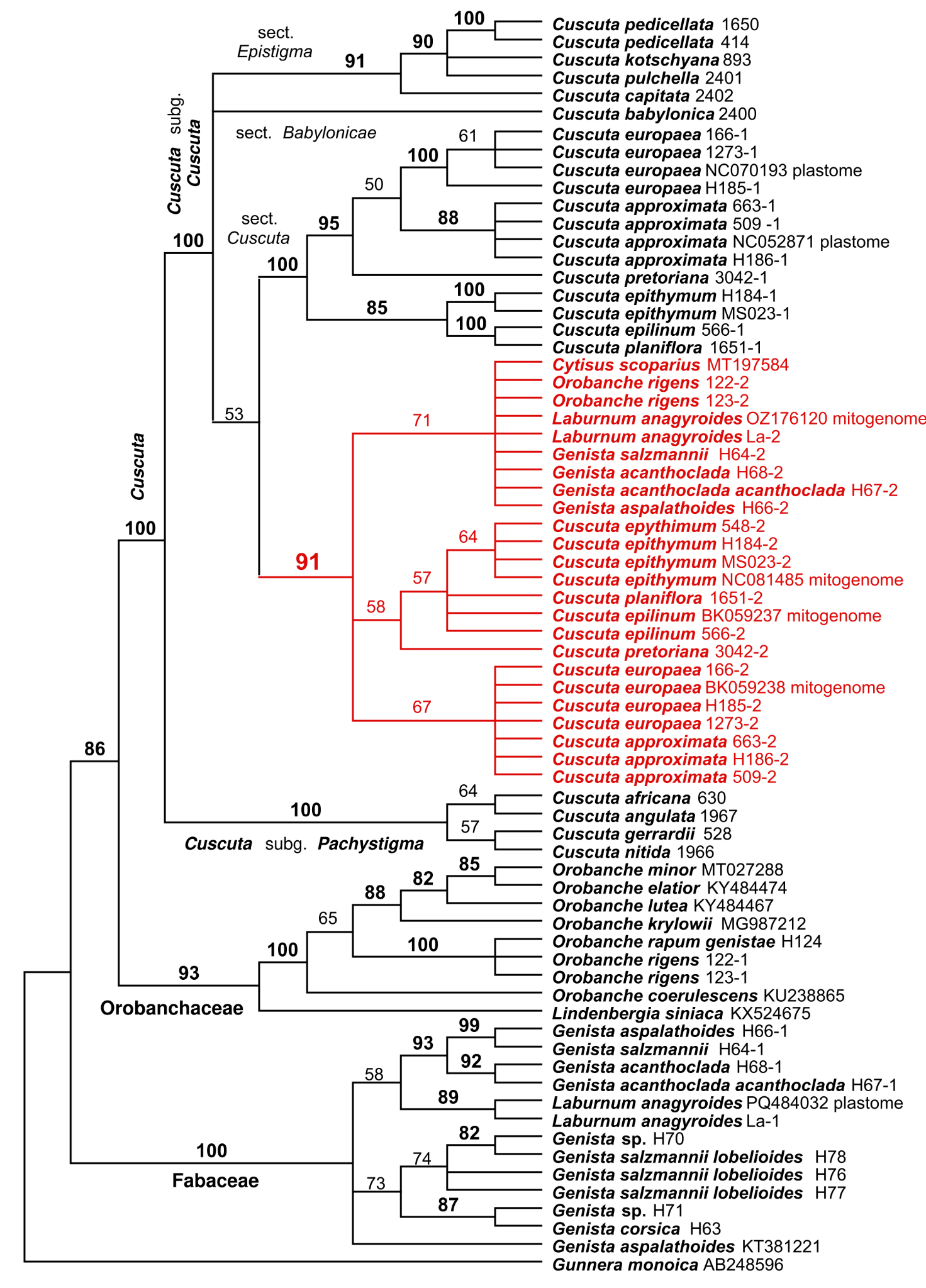


**Supplementary Figure 3.** Bootstrap consensus tree resulting from 500 heuristic bootstrap replicates under maximum parsimony optimality criterion (see text for details of MP analyses and search parameters used). Bootstrap values are indicated for nodes supported at ≥50%. Highlighted in bold are those that are ≥80%. Species names are followed by their DNA accession numbers (Supplementary Table 3). Suffixes -1 and -2 refer to those sequences inferred to be from plastid or mitochondrial genomes, respectively. ‘Plastome’ and ‘mitogenome’ designate *trnL-trnF* sequences extracted from the entire plastid and mitochondrial genome sequences as deposited in GenBank. Compare with Fig. 1 for further details and interpretations.


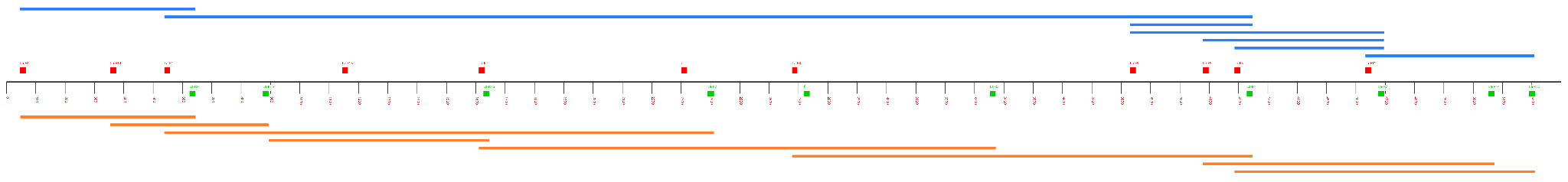


**Supplementary Figure 4.** Primers used in the experiment and PCR products obtained. As reference, region 388200-393500 of the *Laburnum anagyroides* (acc. no. OZ176120) mitochondrial genome assembly sequence was used. Color coding: red - forward primers; green - reverse primers; blue - *Orobanche rigens* PCR products; orange - *Genista salzmannii* products.

## Tables

**Supplementary Table 1.** Top eight results (100%-99% query cover) of the BLAST search for the investigated 420 bp sequence of *Orobanche rigens* (query).

| **Subject** | **Species** | **Query Cover** | **% ident.** | **Accession no.** |
| --- | --- | --- | --- | --- |
| mitochondrion, complete genome | *Laburnum anagyroides* | 100% | 100.00 | OZ176120 |
| chloroplast, *trnL-trnF* region | *Cytisus scoparius* (*Cuscuta* environmental sample)* | 100% | 99.50 | MT197584 |
| mitochondrion, complete genome | *Cuscuta epithymum* | 100% | 91.25 | NC_081485 |
| mitochondrion, complete genome | *Cuscuta epilinum* | 100% | 88.76 | BK059237 |
| chloroplast, complete genome | *Cuscuta approximata* | 100% | 81.59 | NC_052871 |
| chloroplast, *trnL-trnF* region | *Cuscuta approximata* | 100% | 81.59 | EF202557 |
| chloroplast, complete genome | *Cuscuta europaea* | 100% | 79.51 | NC_070193 |
| mitochondrion, complete genome | *Cuscuta europaea* | 99% | 89.43 | BK059238 |

* see text for explanation

**Supplementary Table 2.** List of plant samples used in the study and the corresponding sequence records. Country, locality details, year, collectors, and herbaria in which the specimens are deposited are indicated for all specimens. In addition, DNA extraction and GenBank accession numbers (plastid- and mitochondrion-derived *trnL-trnF* sequences, where available) are provided (compare with Fig. 2). Newly generated sequences are indicated by asterisks. Abbreviations of herbaria follow Index Herbariorum (https://sweetgum.nybg.org/science/ih/).

| **DNA accessions** | **Species** | **Provenance** | **Collected by;**  **Voucher; Herbarium** | **Identified by** | **Year collected** | **Genbank accession numbers** |
| --- | --- | --- | --- | --- | --- | --- |
| Fabaceae (tribe Genisteae) | | | | | | |
| n/a | *Cytisus scoparius*^1^ | Genbank (UK) | unknown | unknown | unknown | n/a; MT197584 |
| H68 | *Genista acanthoclada* | Greece, Rhodes, Poliraki | Stuchlik; 0313614; KRA | L. Stuchlik | 2006 | PV940720*; PX020895* |
| H67 | *Genista acanthoclada* subsp. *acanthoclada* | Greece, Crete, Gournes, N 35° 19’58,6’’, E 025°, 16’, 57,6’ | Zając et al.; 0422635; KRA | A. Zając | 2013 | PV940719*; PX020897* |
| H66 | *Genista aspalathoides* | Italy, Sardinia | Zając, Zdebska; 0324031; KRA | L. Stuchlik | 2006 | PV940721*; PX020894* |
| n/a | *Genista aspalathoides* | Genbank (Algeria) | MARS03815 | unknown | unknown | KT381221; n/a |
| H63 | *Genista corsica* | France, Corsica, near Tattone  N 42° 10’29’’, E 09°, 09’, 57,3’ | Zemanek et al.; 0362007; KRA | L. Stuchlik | 2008 | PV940728; n/a |
| H64 | *Genista salzmannii* | Italy, Sardinia, Capo Testa  N 41° 14’34,3’’, E 09°, 08’, 48,5’, | Zając et al.; 0467394; KRA | J. Zieliński | 2011 | PV940722*; PX020898* |
| H76 | *Genista salzmannii* subsp. *lobelioides* | France, Corsica, Lac Nino | Bioret; KTC | F. Bioret | 2021 | PV940727*; n/a |
| H77 | *Genista salzmannii* subsp. *lobelioides* | France, Corsica, l’Ospédale | Bioret; KTC | F. Bioret | 2021 | PV940726*; n/a |
| H78 | *Genista salzmannii* subsp. *lobelioides* | France, Corsica, du Coscione | Bioret; KTC | F. Bioret | 2021 | PV940725*; n/a |
| H70 | *Genista* sp. | France, Corsica, Ostriconi | Piwowarczyk; KTC | R. Piwowarczyk | 2008 | PV940724*; n/a |
| H71 | *Genista* sp. | Italy, Sardinia, Gennargentu | Piwowarczyk; KTC | R. Piwowarczyk | 2011 | PV940723*; n/a |
| La | *Laburnum anagyroides* | Poland, Market - Szkółka Pnączy Wędrowski, Kolincz | Kwolek, Góralski; KTC | R. Piwowarczyk | 2024 | PV940718*; PX020900* |
| n/a | *Laburnum anagyroides* | Genbank (unknown) | unknown | unknown | unknown | n/a; OZ176120 |
| n/a | *Laburnum anagyroides* | Genbank (unknown) | unknown | unknown | unknown | PQ484032; n/a |
| Orobanchaceae | | | | | | |
| n/a | *Lindenbergia siniaca* | Genbank (Israel) | ERE:79346 | unknown | unknown | KX524675; n/a |
| n/a | *Orobanche elatior* | Poland | Smoczyk; KTC | R. Piwowarczyk | 2014 | KY484474; n/a |
| n/a | *Orobanche coerulescens* | Poland | Piwowarczyk; KTC | R. Piwowarczyk | 2013 | KU238865; n/a |
| n/a | *Orobanche krylowii* | Russia, Irkutsk | Ivanova s.n.; IRKU | R. Piwowarczyk | 1990 | MG987212; n/a |
| n/a | *Orobanche lutea* | Poland | Piwowarczyk; KTC | R. Piwowarczyk | 2013 | KY484467; n/a |
| n/a | *Orobanche minor* | Georgia | Piwowarczyk; KTC | R. Piwowarczyk | 2014 | MT027288; n/a |
| 124 | *Orobanche rapum-genistae* | Spain, Andalusia, El Rocío | Piwowarczyk; KTC | R. Piwowarczyk | 2012 | MF964232; n/a |
| 122 | *Orobanche rigens* | France, Corsica, Ostriconi | Piwowarczyk; KTC | R. Piwowarczyk | 2008 | PV940729*; PX020896* |
| 223 | *Orobanche rigens* | Italy, Sardinia, Gennargentu | Piwowarczyk; KTC | R. Piwowarczyk | 2011 | PV940730*; PX020899* |
| Convolvulaceae | | | | | | |
| 630 | *Cuscuta africana* | South Africa, Western Cape | Oliver 11852; SANBI | S. Stefanović | 2001 | PX514528*; n/a |
| 1967 | *Cuscuta angulata* | South Africa, Western Cape | Stefanović SS-17-134; TRTE | S. Stefanović | 2017 | PX514529*; n/a |
| 663 | *Cuscuta approximata* | Canada, British Columbia | Lomer 92-306; UBC | S. Stefanović | 1992 | PX514527*; PX514539* |
| 509 | *Cuscuta approximata* | Canada, British Columbia | Lomer 93-204; UBC | S. Stefanović | 1993 | EF202557; PX514540* |
| H186 | *Cuscuta approximata* | Georgia, Davit Gareja steppe | Piwowarczyk; KTC | R. Piwowarczyk | 2014 | PV940716*; PX020892* |
| n/a | *Cuscuta approximata* | USA, Utah | Stefanović SS-09-47; TRTE | M. Costea | 2009 | NC052871; n/a |
| 2400 | *Cuscuta babylonica* | Turkmenistan | Barai s.n.; LE | S. Stefanović | 1952 | PX514521*; n/a |
| 2402 | *Cuscuta capitata* | Pakistan, Khyber-Pakhtunkhwa | Wendelbo s.n.; LE | S. Stefanović | 1950 | PX514520*; n/a |
| 566 | *Cuscuta epilinum* | Canada, Quebec | Cartier s.n.; DAO | S. Stefanović | 1941 | PX514522*; PX514535* |
| n/a | *Cuscuta epilinum* | Genbank (unknown) | unknown | unknown | unknown | n/a; BK059237 |
| MS-023 | *Cuscuta epithymum* | Serbia | Šarić MS-023; no voucher | M. Šarić | 2013 | KC569804; PX514533* |
| H184 | *Cuscuta epithymum* | Poland, Kików | Piwowarczyk; KTC | R. Piwowarczyk | 2016 | PV940717*; PX020893* |
| 548 | *Cuscuta epithymum* | New Zealand, Canterbury | Cummings 260526; CHR | S. Stefanović | 1973 | n/a; PX514532 |
| n/a | *Cuscuta epithymum* | Genbank (unknown) | unknown | unknown | unknown | n/a; NC081485 |
| 166 | *Cuscuta europaea* | Finland, Uusimaa | Alanko 94416; H | S. Stefanović | 1997 | PX514525*; PX514537* |
| 1273 | *Cuscuta europaea* | Finland, Uusimaa | Stefanović SS-11-30; TRTE | S. Stefanović | 2011 | PX514526*; PX514538* |
| H185 | *Cuscuta europaea* | Georgia, Bodbe | Piwowarczyk; KTC | R. Piwowarczyk | 2014 | PV940715*; PX020891* |
| n/a | *Cuscuta europaea* | Genbank (Bulgaria) | unknown | unknown | unknown | NC070193; n/a |
| n/a | *Cuscuta europaea* | Genbank (unknown) | unknown | unknown | unknown | n/a; BK059238 |
| 528 | *Cuscuta gerrardii* | South Africa; Mpumalanga | Burrows 4666; J | S. Stefanović | 1989 | PX514530*; n/a |
| 893 | *Cuscuta kotschyana* | Iran, Mazandaran | Alava10608; RSA | S. Stefanović | 1972 | PX514518*; n/a |
| 1966 | *Cuscuta nitida* | South Africa, Western Cape | Stefanović SS-17-144; TRTE | S. Stefanović | 2017 | PX514531*; n/a |
| 414 | *Cuscuta* *pedicellata* | Saudi Arabia | Humbles 10061; IND | S. Stefanović | 1978 | PX514517*; n/a |
| 1650 | *Cuscuta* *pedicellata* | Palestine/Israel | Musselman 10412; ODU | S. Stefanović | 1987 | PX514516*; n/a |
| 1651 | *Cuscuta planiflora* | Palestine/Israel | Musselman 10392; ODU | S. Stefanović | 1987 | PX514523*; PX514534* |
| 3042 | *Cuscuta pretoriana* | South Africa, Gauteng | Stefanović SS-24-10; TRTE | S. Stefanović | 2024 | PX514524*; PX514536* |
| 2401 | *Cuscuta pulchella* | Tajikistan | s.col. 5445; LE | S. Stefanović | 1973 | PX514519*; n/a |
| Gunneraceae | | | | | | |
| n/a | *Gunnera monoica* | Genbank (New Zealand, Otago) | MAK:MAK358964 | unknown | unknown | AB248596; n/a |

NB: ^1^ This sample in GenBank is deposited as “*Cuscuta* environmental sample isolate EDNA16-0043485 *trnL-trnF* intergenic spacer region, partial sequence; chloroplast”, with a note that further explains that “sample purchased as *Cytisus scoparius*, Broom by TGoNS, 2016”.

**Supplementary Table 3.** Primers used for amplifications and/or sequencing of the *Pytheas* sequence.

| **name** | **sequence** |
| --- | --- |
| *La-F1* | GACTATTCGGGGGAGCTGGA |
| *La-F2* | ATTACCCAATCACACCCCGA |
| *La-F3* | GGTCGGGATAGCTCAGCTG |
| *La-F4* | AACTGGCTTCCATACTGGGC |
| *La-F5* | CCCCATTCCCCCTTGAAGAC |
| *La-F8* | GGACTAGATCGAAAGCCCGG |
| *La-F9* | AGGGAAGGGTTAATGACGCG |
| *La-F11* | AGACAGAATCCAAAGCCGCT |
| *La-F14* | AAGCTACTGCCCTTGACAGC |
| *La-F15* | ACGTCAATGATGCTGCAACC |
| *La-R1* | CCTCTATCTCAAAACCCCCGG |
| *La-R2* | AGGTTTTTGGGGATAGAGGGAC |
| *La-R3* | TGCCCTAACTTAACCTGCCC |
| *La-R4* | TCACCCACTCCGCCAAATAC |
| *La-R7* | CGCCTTCCATTCCGTACCTT |
| *La-R13* | CCAGTCTTTGAGGTCGCCTT |
| *La-R14* | GTGTAAACTTGACGCGCTGA |
| *La-R15* | GGCGGATACTTCTGATCCCG |
| *La-R16* | TCTACAGCTGTGGCAATCGG |
| *c** | CGAAATCGGTAGACGCTACG |
| *f** | ATTTGAACTGGTGACACGAG |

NB: *primers from [^34^](https://www.zotero.org/google-docs/?IUe6oa)
